# *Endarachne binghamiae* Extract Ameliorates Inflammatory Responses in Macrophages Through Regulation of MAPK, NF-kB and PI3K/AKT Pathways, and Prevents Acute Lung Injury in Mice

**DOI:** 10.3390/life15010088

**Published:** 2025-01-13

**Authors:** Sang-Hoon Lee, Sang-Seop Lee, Ga-Young Lee, Seung-Yun Han, Dong-Sub Kim, Bong-Ho Lee, Yung-Choon Yoo

**Affiliations:** 1 Department of Microbiology, College of Medicine, Konyang University, Daejeon 32992, Republic of Korea; parkss2105@naver.com (S.-H.L.); wgd.aria@gmail.com (S.-S.L.); jc0012003@naver.com (G.-Y.L.); 2 Department of Anatomy, College of Medicine, Konyang University, Daejeon 32992, Republic of Korea; jjzzy@konyang.ac.kr; 3 Division of Natural Product Research, Korea Prime Pharmacy Co., Ltd., Gwangju 58144, Republic of Korea; ds.kim@koreaprime.co.kr; 4 Department of Chemical Technology, Hanbat National University, Daejeon 34158, Republic of Korea; ibh011@hanbat.ac.kr

**Keywords:** *Endarachne binghamiae*, inflammation, acute lung injury, MAPK, NF-kB

## Abstract

In this study, the anti-inflammatory effect of the hot water extract of Endarachne binghamiae (EB-WE), a type of marine brown algae, was investigated in LPS-stimulated RAW 264.7 cells and an acute lung injury (ALI) mouse model induced by intranasal LPS administration. Treatment with EB-WE significantly inhibited NO and pro-inflammatory cytokine (TNF-a and IL-6) production in LPS-stimulated RAW 264.7 cells. In mRNA analysis, the expression of pro-inflammatory cytokines, COX-2, and iNOS mRNAs, was down-regulated by EB-WE treatment. The phosphorylation of MAPK, IkB, and PI3K/AKT molecules responsible for signal pathways during inflammation in LPS-stimulated macrophages was also significantly inhibited by EB-WE. In an in vivo model for ALI, oral administration of EB-WE significantly reduced the level of pro-inflammatory cytokines (TNF-a, IL-1b, and IL-6) and chemokines (MCP-1, CXC-16, CXCL1, and TARC) in serum or bronchoalveolar lavage fluid (BALF) of mice. Similarly to the results in LPS-stimulated RAW 264.7 cells, treatment with EB-WE significantly inhibited intracellular signal pathways mediated by MAPK, IkB, and PI3K/AKT in lung tissues of mice with ALI, and also decreased the expression of mRNAs of inflammatory mediators such as TNF-a, IL-6, iNOS, and COX-2. Furthermore, the inhibitory effect of EB-WE on ALI was apparently confirmed in histological examination through lung tissue staining. Taken together, it is clear that EB-WE has potential activity to effectively ameliorate the inflammatory responses in macrophages through down-regulation of MAPK, NF-kB, and PI3K/AKT activation, and suppress acute lung injury induced by LPS. These findings strongly suggest that EB-WE is a promising natural product beneficial for developing preventive treatments and cures of inflammation-related diseases.

## 1. Introduction

Inflammation is directly related to numerous health issues [1]. Among these, respiratory function is fundamental to maintaining homeostasis in living organisms as the respiratory system is the primary organ in contact with the external environment [2]. Consequently, the respiratory system is constantly exposed to airborne pathogens, and the immune response of respiratory epithelial tissues is regularly activated to combat these challenges [3]. Despite the body’s efforts, inflammation of the respiratory tract occurs with considerable frequency, the main cause of which is infection with Gram-negative bacteria. The inflammatory response triggered by these bacteria can occur very rapidly, and certain immune cells require a relatively longer time to remove antigens from lung tissue [4]. Therefore, lung infections with Gram-negative bacterial often lead to severe lung damage, known as acute lung injury (ALI), a serious condition associated with a high mortality rate due to complications such as sepsis [5]. The risk of this condition has increased due to the negative synergy with modern-day air pollution and climate change [6].

Among immune cells, macrophages and dendritic cells, known as antigen-presenting cells (APCs), play important roles in initializing immune responses, processing antigen molecules, and presenting antigenic peptides to T lymphocytes [7]. Lipopolysaccharide (LPS), an endotoxin derived from Gram-negative bacteria, binds mainly to toll-like receptor 4 (TLR4) on the surface of these cells and induces inflammatory responses via the MyD88-mediated pathway [8]. This process leads to a strong Th1 immune response, driven by the release of pro-inflammatory cytokines and the expression of TLR co-stimulatory molecules [9]. While the cytokines and chemokines secreted in response to TLR4 activation are essential for immune activation and the production of antibodies necessary to fight infection, excessive activation of these immune cells can result in severe inflammatory conditions such as sepsis and tissue injury [10]. Therefore, regulating excessive immune responses in macrophages and dendritic cells has emerged as a critical issue for controlling inflammatory diseases [11].

*Endarachine binghamiae* (EB), a brown algae, is primarily found in many coastal areas such as Western United States, the Republic of Korea, and Japan. Traditionally, this alga is used as an edible seaweed in many countries around the world [12]. EB has morphological characteristics of having leaves measuring approximately 25–30 mm wide and 250–300 mm tall [13]. EB is known to contain mannitol, fucoidan, laminarin, alginic acid, and other components beneficial for immune regulation [14]. In a previous study, we demonstrated that EB-WE administration improved ischemic brain injury induced by carotid artery occlusion in rats through the upregulation of NRF-2 (nuclear factor erythroid 2-related factor 2) and HO-1 (heme oxygenase-1) expression [15]. The LC-MS analysis conducted in this study confirmed that EB-WE contains several active compounds, including five major components: kansuinin D, acanthoside D, saikosaponin E, bacanoic acid methyl ester, and (R)-ricinoleic acid.

Previous studies showed that EB has beneficial properties including anti-diabetic and anti-oxidant activities [16,17,18]. In one of these studies, the most recent study reported the activity of EB to protect murine myoblast cells against oxidative stress through upregulating the expression of anti-oxidant enzyme like HO-1 and transcriptional regulator like Nrf2 [17]. However, there are few reports on not only the intracellular mechanisms involved in EB’s anti-inflammatory activity but also preventive effects on inflammatory diseases like acute lung injury.

In this study, we investigated the anti-inflammatory effects of EB-WE on LPS-stimulated RAW 264.7 macrophages and related intracellular mechanisms, and evaluated preventive effects on LPS-induced acute lung injury in mice.

## 2. Materials and Methods

### 2.1. Preparation of EB-WE

EB-WE was prepared by the method described previously [15]. EB was harvested from Seogwipo-si, Jeju Island, in May 2021. Fifty grams of EB were finely ground, mixed with 500 mL of distilled water, and extracted at 80 °C for 6 h. The extract was filtered using Whatman filter paper No. 4, and the filtered solution was centrifuged at 2250× *g* for 10 min. After centrifugation, the supernatant was dried using a freeze dryer (VaCo2, Niedersachsen, Germany) at −83 °C. The hot water extract of EB (EB-WE) was stored at −30 °C until use, with a total yield of 14.46%.

### 2.2. Cell Culture and Cytotoxicity Assay

The RAW 264.7 cells (Korean Cell Line Bank, Seoul, Republic of Korea) were cultured in Dulbecco’s Modified Eagle’s Medium (DMEM; Gyeongsan, Gyeongsangbuk-do, Republic of Korea) supplemented with 10% fetal bovine serum (FBS; Waltham, MA, USA) and 1% antibiotics (penicillin–streptomycin; Sigma-Aldrich Co., St. Louis, MO, USA) at 37 °C in a humidified atmosphere with 5% CO_2_. The cytotoxicity of EB-WE on the RAW 264.7 cells was assessed using the MTT assay. The RAW 264.7 cells (5 × 10^4^/well) were placed for 8 h, and then incubated with EB-WE at concentrations ranging from 0 to 500 µg/mL for 12 h. Subsequently, LPS (*E. coli* serotype 055, Sigma-Aldrich Co.) was added at a concentration of 1 µg/mL to induce an inflammatory response for 24 h. The MTT solution (5 mg/mL; Sigma-Aldrich Co.) was then added, and the cells were incubated for 2 h. After centrifugation (1500 rpm/5 min), the supernatant was removed, and the resulting formazan was dissolved in dimethyl sulfoxide. The absorbance was measured at 540 nm using a microplate reader (ELx 800-PC; BioTek Instruments Inc., Winooski, VT, USA).

### 2.3. Determination of NO, Cytokines, and Chemokines

The level of NO production in the supernatants of the RAW 264.7 cells was determined by adding a Griess reagent (40 mg/mL; Sigma-Aldrich Co.) to the culture medium. The mixture of cell culture medium (50 µL) and Griess reagent (50 µL) was incubated at room temperature, and absorbance was measured at 540 nm using a microplate reader. The level of TNF-α and IL-6 in the cell cultures was quantified using ELISA kits (BD Bioscience, San Jose, CA, USA) according to the manufacturer’s indication. The level of cytokines and chemokines in the sera and bronchoalveolar lavage fluid (BALF) of the mice with ALI induced by LPS administration was also determined using ELISA kits (BD Bioscience).

### 2.4. Real-Time PCR Analysis

The RAW 264.7 cells (1 × 10^6^ cells/well) were seeded in 6-well plates and incubated under the same conditions with the above experiments. Eight hours after LPS treatment, the RNA of the cells was isolated using a TRIzol reagent (iNtRON Biotechnology, Seongnam, Gyeonggi-do, Republic of Korea), and its amount was quantified by spectrophotometry. cDNA was synthesized using the quantified RNA by a power cDNA synthesis kit (iNtRON Biotechnology), and was subsequently subjected to real-time PCR using Cfx96 (Bio-Rad, Hercules, CA, USA). The analysis of mRNA expression in the lung tissues of the mice with ALI was conducted as the same method with that of the RAW 264.7 cells. The primers for PCR analysis were purchased from CosmoGentech (Seoul, Republic of Korea), and DNA polymerase was purchased from Bio-Rad (Hercules, CA, USA). The sequences of all PCR primers are as shown in Table 1.

### 2.5. Western Blot for Signal Transduction Assay

The RAW 264.7 cells (1 × 10^6^ cells/well) were pre-treated with 100 or 200 mg/mL of EB-WE and followed by LPS stimulation. Fifteen minutes after LPS stimulation, the cells were washed three times with cold PBS and lyzed with lysis buffer (RIPA buffer, protease, and phosphatase inhibitor cocktail, Thermo Scientific, Waltham, MA, USA). Cellular proteins were obtained from the precipitant after centrifugation (14,000 rpm, 10 min, 4 °C), and their concentrations were quantified using a BCA protein assay kit (Sigma Aldrich Co.). Thereafter, the protein (20 mg) was electrophoresed on 10% sodium dodecyl sulfate-polyacrylamide gel electrophoresis (SDS-PAGE), and the gel was transferred to PVDF membranes (polyvinylidene difluoride membrane, Bio-Rad, Hercules, CA, USA) for 2 h. The membranes were then blocked with 5% skim milk (BD Bioscience, San Jose, CA, USA) for 1 h and reacted with each of the antibodies (rabbit monoclonal antibody, BD Bioscience, Franklin Lake, NJ, USA) specific to p-IkB, IkB, p-JNK, JNK, p-ERK, ERK, p-p38, p38, p-AKT, AKT, p-PI3K, or PI3K. The membranes were then washed eight times with TBST (1xTBS, 0.05% Tween20; Sigma Aldrich Co.) and reacted with horseradish peroxidase (HRP)-conjugated anti-rabbit IgG (BD Bioscience), a secondary antibody against each antibody. After washing with TBST eight times, the membranes were analyzed using a chemiluminescence imaging system. The analysis of intracellular proteins associated with LPS-induced signal transduction in the lung tissues was performed using the same protocol as above. The protein content of the lung tissues used in Western blot was 30 mg per group.

### 2.6. Animal Experiment for ALI

Seven-week-old male BALB/c mice (19–22 g) were purchased from Raon Bio (Yongin, Republic of Korea). The mice were housed and maintained in accordance with the guidelines approved by the Animal Ethics Committee of Konyang University (Approval No. P-23-22-A-01). For the ALI experiment, the mice were randomly divided into five groups with seven mice per group: the normal group, LPS group, Dexamethasone (Sigma Aldrich Co.) group (75 mg/mouse), EB-WE 2 mg/mouse group, and EB-WE 5 mg/mouse group. Dexamethasone (positive control) and EB-WE were administered orally (p.o.) once daily for three days before LPS administration. ALI was induced by intranasal (i.n.) administration of LPS (100 µg/mouse). The mice were sacrificed 18 h after LPS administration, and lung tissues, blood, and bronchoalveolar lavage fluid (BALF) were collected for analysis.

### 2.7. Body Temperature and Lung Edema Measurement

Twenty-four hours after LPS administration, the body temperature of the mice was measured using a thermometer. For the lung edema assessment, the lungs were weighed to measure the wet weight. The lungs were subsequently dried in an oven at 60 °C for 48 h to determine the dry weight. The wet/dry ratio was calculated to assess the degree of lung edema induced by LPS.

### 2.8. Histology

Lung tissues isolated from the mice with ALI were fixed with 4% paraformaldehyde (Image-IT™ Fixative Solutions; Invitrogen, Waltham, MA, USA), dehydrated with a 30% sucrose (MB-S4842; MBCELL, Seoul, Republic of Korea) solution, and embedded with Tissue-Tek OCT compound (Sakura Finetek USA Inc., Torrance, CA, USA). Frozen sections made with a cryosectioning machine (CM1520; Leica Biosystems Nussloch GmbH, Nußloch, Germany) were stained with Hematoxylin and Eosin (H&E) to compare immune cell infiltration in the lung tissues.

### 2.9. Statistical Analysis

Statistical analysis was performed using IBM SPSS 25 and SAS 9.4, and significance was assessed using Student’s *t*-test.

## 3. Results

### 3.1. EB-WE Inhibits Production of Inflammatory Mediators in LPS-Stimulated RAW 264.7 Cells

**Figure 1:**
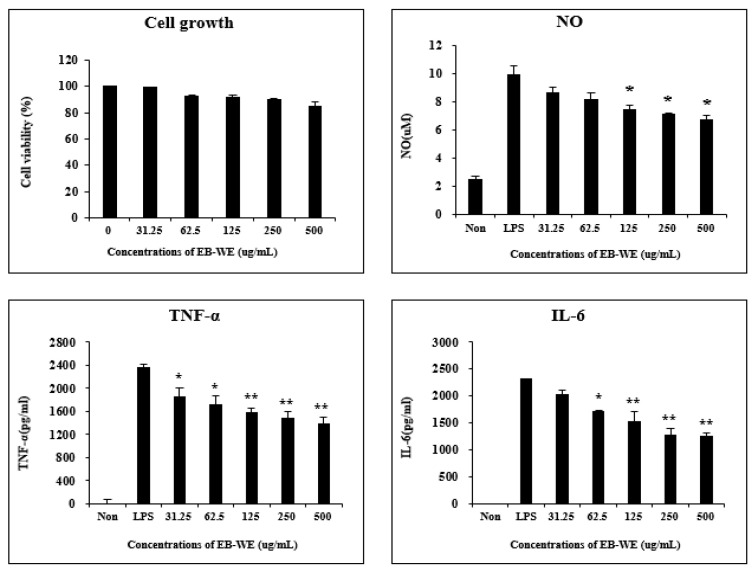
EB-WE inhibits the production of inflammatory mediators in LPS-induced RAW 264.7 macrophages. Cell viability was measured by MTT assay. Level of NO and pro-inflammatory cytokines (TNF-α and IL-6) was determined using Griess reagent and ELISA kits, respectively. * *p* < 0.05; ** *p* < 0.01, compared to LPS-stimulated group by Student’s two-tailed *t*-test.

Safe concentrations of EB-WE for RAW 264.7 macrophages were determined using the MTT assay. EB-WE was shown to have no cytotoxicity up to the concentration of 500 μg/mL (Figure 1). Based on these results, subsequent experiments were conducted at concentrations below 500 mg/mL. When the RAW 264.7 cells were stimulated with LPS, they produced NO as well as pro-inflammatory cytokines such as TNF-α and IL-6. These cytokines are predominantly produced by macrophages and play a key role in amplifying the inflammatory response and promoting systemic inflammation [19]. Therefore, controlling the secretion of these inflammatory mediators is an essential action for anti-inflammatory activity. Treatment with EB-WE inhibited the production of TNF-α, IL-6, and NO in LPS-stimulated RAW 264.7 cells, showing potential anti-inflammatory activity (Figure 1).

### 3.2. Inhibition of mRNA Expression of Inflammatory Mediators by EB-WE

**Figure 2:**
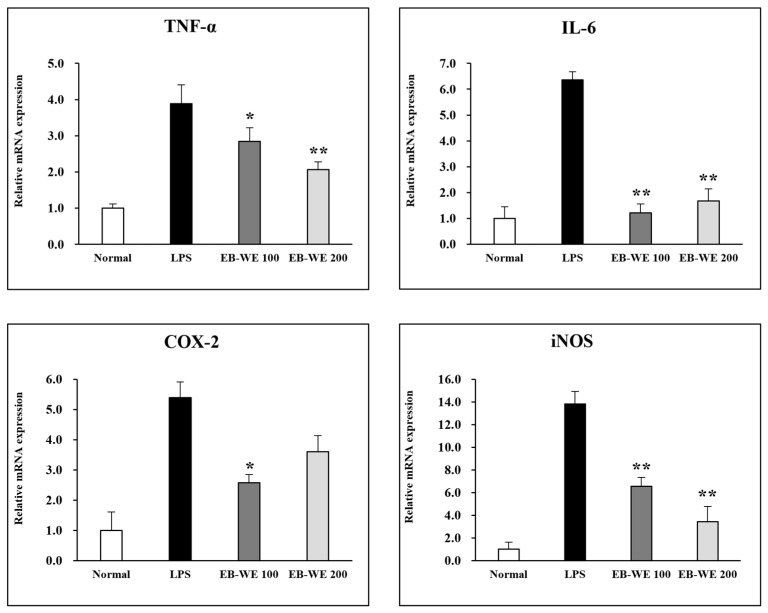
EB-WE inhibits expression of mRNAs of iNOS, TNF-α, IL-6, and COX-2 in LPS-stimulated RAW 264.7 cells. * *p* < 0.05; ** *p* < 0.01, compared to LPS-stimulated group by Student’s two-tailed *t*-test.

The expression of mRNAs of inflammatory mediators in LPS-stimulated RAW 264.7 cells was evaluated by real-time PCR analysis. Treatment with EB-WE significantly suppressed the expression of mRNAs of iNOS (an enzyme that mediates NO production), TNF-α, and IL-6 (Figure 2). Furthermore, EB-WE was shown to inhibit the expression of COX-2 mRNA, an enzyme that converts arachidonic acid into prostaglandin (PGF-2) involved in the induction of inflammation [20]. 

### 3.3. Signal Transduction Inhibition of EB-WE in LPS-Stimulated RAW 264.7 Cells

**Figure 3:**
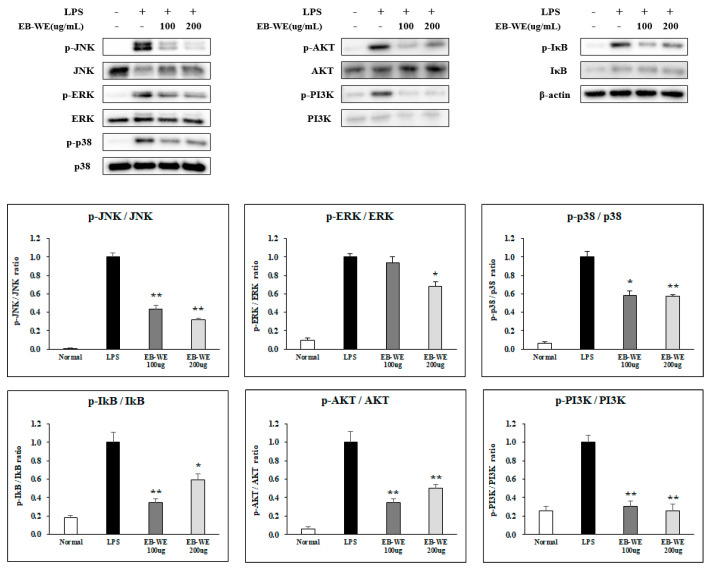
EB-WE suppresses signal pathways mediated by MAPKs, NF-kB, and PI3/AKT in LPS-stimulated RAW 264.7 cells. Phosphorylation of TLR4-mediated signaling molecules was analyzed by Western blot. * *p* < 0.05; ** *p* < 0.01, compared to group stimulated with LPS by Student’s two-tailed *t*-test.

During the process of inflammation in LPS-stimulated macrophages, many types of intracellular signal transduction pathways including MAPK, NF-κB, and PI3/AKT are activated to produce inflammatory mediators [20,21]. In order to determine which signal transduction system is regulated by EB-WE in suppressing macrophage inflammatory responses, the phosphorylation of intracellular signaling molecules mediated by TLR4 was investigated. Treatment with EB-WE significantly inhibited the phosphorylation of MAPKs (ERK, JNK, and p38), I-κB, and PI3K/AKT (Figure 3). These findings suggest that EB-WE exerts anti-inflammatory effects by suppressing the activation of MAPK, NF-κB, and PI3K/AKT signaling pathways.

### 3.4. EB-WE Suppresses Body Temperature and Lung Edema in ALI In Vivo Model

**Figure 4:**
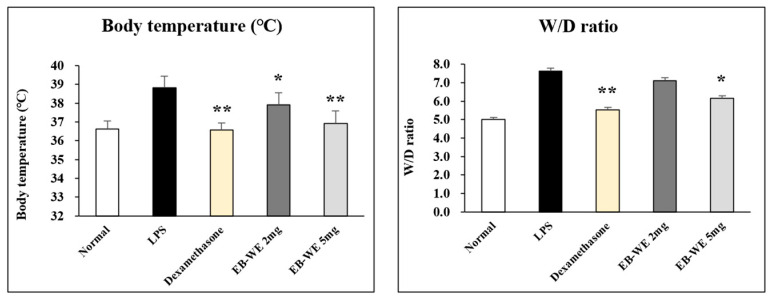
Oral administration of EB-WE inhibits elevated body temperature and pulmonary edema in ALI model. * *p* < 0.05; ** *p* < 0.01, compared to group administered with LPS by Student’s two-tailed *t*-test.

Next, to address whether the anti-inflammatory activity of EB-WE seen in RAW 264.7 macrophages is also effective in inflammation-related diseases, its effect was confirmed in an in vivo ALI model using mice. In lung inflammation such as ALI, fever and pulmonary edema are typically observed symptoms [22]. Oral administration of EB-WE significantly inhibited elevated body temperature and pulmonary edema caused by the inflammatory responses in the mice with ALI (Figure 4). In addition, a high dosage (5 mg/mouse) of EB-WE showed almost the same level of inhibitory effects as the positive control Dexamethasone [23] in suppressing fever and lung edema.

### 3.5. EB-WE Ameliorates Histopathological Changes in ALI Model Lungs

**Figure 5:**
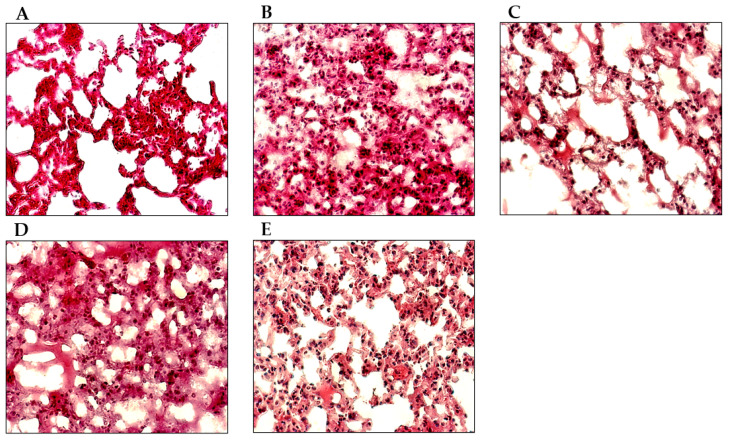
Oral administration of EB-WE alleviated histopathological changes in the lungs of mice with ALI. Histological and pathological assessment of therapeutic potential of EB-WE on LPS-induced ALI in mice was assessed 48 h after LPS challenge by H&E staining. (**A**) Non-treated; (**B**) LPS; (**C**) Dexamethasone; (**D**) EB-WE 2 mg/mouse; (**E**) EB-WE 5 mg/mouse.

To assess the potential function of EB-WE in the histopathological changes in the lungs of the mice with induced LPS, the observation of histological and pathological changes in lung sections was carried out 48 h after the i.n. administration of LPS. The lungs of the LPS-induced ALI mice showed the apparent inflammatory infiltrates, increased thickening of interalveolar septum, and interstitial edema (Figure 5B). Oral administration of EB-WE effectively reduced the airspace inflammation and inflammatory infiltrates (Figure 5D,E).

### 3.6. EB-WE Inhibits Level of Cytokines and Chemokines in BALF and Serum

**Figure 6:**
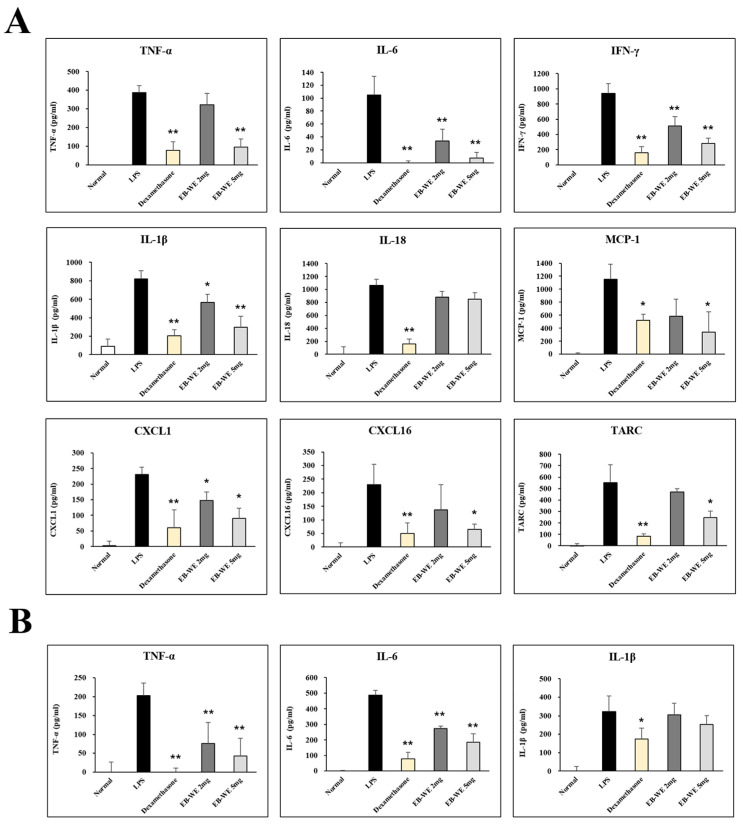
Oral administration of EB-WE ameliorates increase in cytokine and chemokine concentrations in BALF and serum in ALI model. (**A**) Cytokine and chemokine levels of BALF; (**B**) cytokine levels of serum. * *p* < 0.05; ** *p* < 0.01, compared to group administered with LPS by Student’s two-sided *t*-test.

In the ALI model, the intranasal administration of LPS causes inflammation in the lungs, resulting in elevations of the level of pro-inflammatory cytokines and chemokines in BALF and serum [24,25]. This means that the concentrations of pro-inflammatory cytokines and chemokines in BALF and serum are very important indicators for determining the severity and progression of lung injury caused by inflammation. From this perspective, we measured the concentration of various cytokines and chemokines in BALF and serum to determine whether their decrease was correlated with the inhibition of ALI by EB-WE. The oral administration of EB-WE significantly reduced the levels of many types of cytokines (TNF-a, IL-6, IFN-g, IL-1b, IL-8) and chemokines (MCP-1, CXCL16, CXCL1, TARC) in BALF in the mice with ALI (Figure 6A). Additionally, serum levels of TNF-α, IL-6, and IL-1β were also decreased by EB-WE administration (Figure 6B). These results clearly suggest that the potential activity of EB-WE to ameliorate pulmonary inflammation is due to the suppression of the secretion of cytokines and chemokines caused by LPS-induced inflammation in the lungs during ALI.

### 3.7. Regulation of mRNA Expression by EB-WE in Lung Tissues

**Figure 7:**
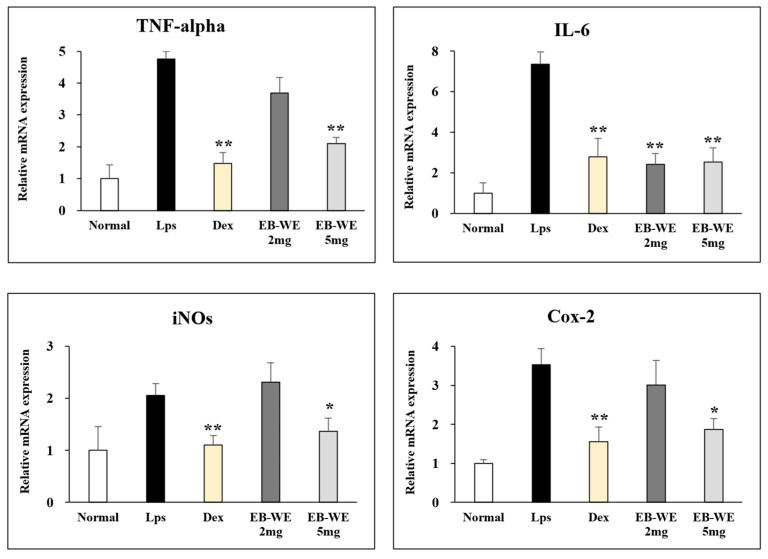
Oral administration of EB-WE inhibits expression of mRNAs for inflammatory mediators in lung tissues in ALI model. * *p* < 0.05; ** *p* < 0.01, compared to group administered with LPS by Student’s two-sided *t*-test.

To examine the regulation of mRNA expression by EB-WE in the ALI model, the expression of mRNAs for pro-inflammatory cytokines (TNF-a and IL-6), iNOS, and COX-2 in the lung tissues was analyzed by real-time PCR. Figure 7 shows that the oral administration of EB-WE apparently down-regulated the expression of mRNAs for all of these inflammatory mediators in the lung tissues in the ALI model. These findings were consistent with the inhibition of the expression of mRNAs for inflammatory mediators in LPS-stimulated RAW 264.7 macrophages, as described in Figure 2.

### 3.8. Inhibition of Signal Transduction by EB-WE in Lung Tissues

**Figure 8:**
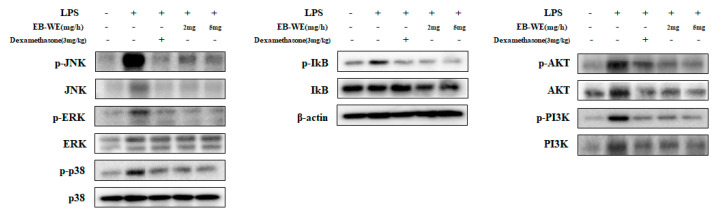

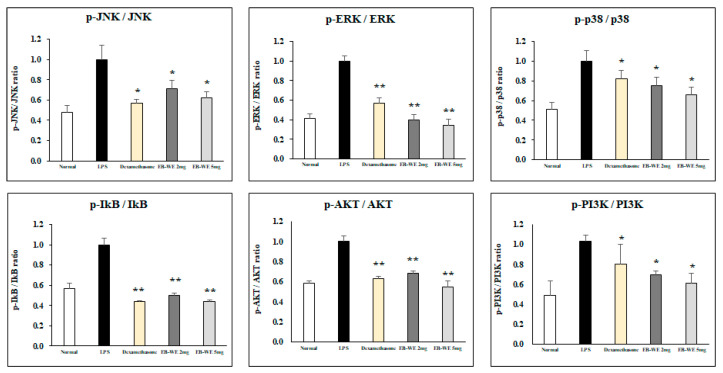
EB-WE showed similar inhibitory effect to dexamethasone on NF-kB, MAPK, and PI3K/AKT pathways in lung tissues. Administration of EB-WE suggests that inflammatory response is suppressed by regulating inflammatory cytokines and activating immune cells through inhibition of signaling pathway, as indicated above. * *p* < 0.05; ** *p* < 0.01 were compared to group stimulated with LPS by Student’s two-sided *t*-test.

In order to investigate the inhibitory effect of EB-WE on lung inflammation at the tissue level, the level of phosphorylation of intracellular signaling mediator proteins involved in inflammation was measured by Western blot assay using lung tissue from the mice administered LPS. Similarly to the results of Figure 3 in which EB-WE inhibited the phosphorylation of MAPKsm, NF-kB, and PI3/AKT molecules in LPS-stimulated RAW 264.7 macrophages, the oral administration of EB-WB significantly reduced the phosphorylation of intracellular proteins associated with the TLR4-mediated inflammatory signal pathways in the lung tissues (Figure 8). These results suggest that EB-WE exerts its protective effects against ALI via inhibiting the activation of key signal pathways mediated by MAPKs, NF-κB, and PI3K/AKT, which induce macrophage activation in response to external stimuli [26], and down-regulating the secretion of pro-inflammatory cytokines and chemokines.

## 4. Discussion

**Figure 9:**
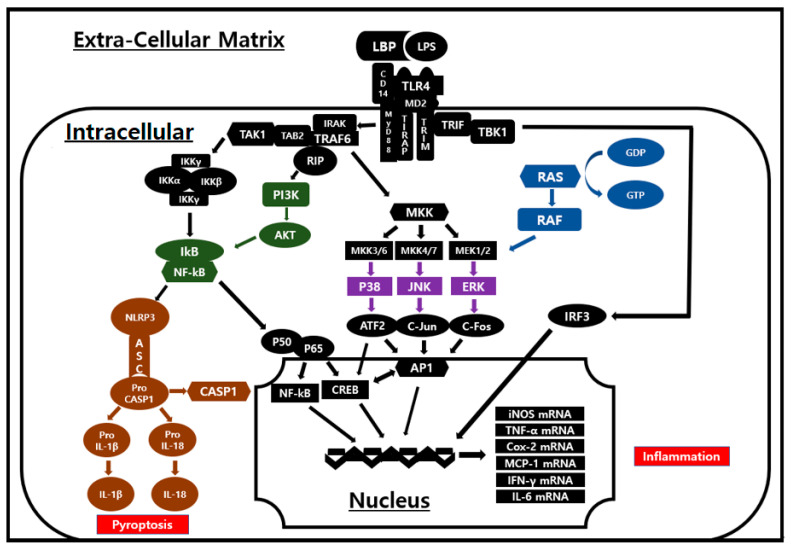
Schematic representation of TLR4 inflammatory pathway. When LPS, bound by LBP, interacts with TLR4, phosphorylation of MAPK occurs through MyD88-dependent pathway. TAK1 and AKT phosphorylate IκB, leading to nuclear translocation of NF-κB. This process activates transcription factors such as AP-1, ATF-2, and P65 Rela in nucleus, which induce expression of inflammatory mediators including TNF-α, IL-6, iNOS, and COX-2. These mediators subsequently drive inflammatory response.

The MAPK signaling pathway plays a central role in regulating cellular responses to various external stimuli and is composed of key kinases, such as ERK, JNK, and p38, which are mediators of inflammatory responses [27]. NF-κB is a transcription factor involved in the production of inflammatory cytokines and chemokines, as well as cell survival, in response to external stimuli like LPS. Under normal conditions, NF-κB is bound to IκB in an inactive state in the cytoplasm. Upon external stimulation, IκB is phosphorylated, allowing NF-κB to translocate to the nucleus where it promotes the expression of inflammatory cytokines [28,29]. In LPS-induced inflammatory response, LPS binds to LBP (LPS binding protein) on the surface of macrophages with the assistance of CD14 and MD2 and transduces activation signals via the cytoplasmic MyD88 molecule [29,30]. Through the MyD88-dependent pathway, the phosphorylation of IκB leads to the nuclear translocation of NF-κB, while the phosphorylation of MAPK activates transcription factors such as CREB and AP-1. These processes ultimately result in the production of mRNA for iNOS, TNF-α, COX-2, MCP-1, IL-6, and others [31,32]. In the TRIF-dependent pathway, mRNA for IFN-γ is generated, which subsequently drives the inflammatory process [30] (Figure 9).

In this study, treatment with EB-WE in LPS-stimulated RAW 264.7 macrophages showed anti-inflammatory activity by inhibiting the phosphorylation of MAPKs (ERK, JNK, p38) and IκB thereby preventing the nuclear translocation of NF-κB (Figure 3). Additionally, it was found that EB-WE modulates the PI3K/AKT pathway. Numerous studies have demonstrated that the PI3K/AKT pathway is crucial for cell differentiation, apoptosis, proliferation, growth, and activation in macrophages, and it acts as an upstream regulator of NF-κB activation [33,34,35,36,37,38]. Therefore, the anti-inflammatory activity of EB-WE, observed in (Figure 1 and Figure 2), is thought to be based on the inhibition of NF-κB and MAPK phosphorylation through the regulation of the PI3K/AKT signaling pathway, leading to the down-regulation of pro-inflammatory cytokines, iNOS, and COX-2 (Figure 3) [20].

In the same context, EB-WE exhibited a potential anti-inflammatory effect on acute lung injury, a critical inflammatory disease in the lung, induced by the intranasal administration of LPS. The oral administration of EB-WE effectively ameliorated inflammatory responses such as elevated body temperature, lung edema, and histopathological changes in the ALI model (Figure 4 and Figure 5). In addition, the mice administered EB-WE orally dramatically decreased the elevated cytokine and chemokine levels in the lungs and sera (Figure 6). The analysis of intracellular proteins related to signal pathways also showed that the oral administration of EB-WE gave rise to the significant inhibition of phosphorylation of MAPK, NF-κB, and PI3K/AKT in the lung tissues in the mice with ALI (Figure 8).

As mentioned earlier, the LC-MS analysis conducted in our previous study identified kansuinin D, acanthoside D, saikosaponin E, bacanoic acid methyl ester, and (R)-ricinoleic acid as the major components of EB-WE. Among these components, acanthoside D was reported to exhibit anti-inflammatory effects against TLR4-mediated pathway activation induced by LPS during a cell-based in vitro experiment as well as in vivo experiment using mice in pneumonia [39,40]. Additionally, (R)-ricinoleic acid has shown edema-reducing and analgesic effects in carrageenan-induced mice edema models [41,42]. Considering these findings, it is possible that the inhibitory effect of EB-WE on TLR4-mediated inflammatory responses observed in this study is associated with the action of these two key components and other active ingredients. Currently, we are conducting additional research to investigate the anti-inflammatory activity of each active component of EB-WE.

The present study suggests that EB-WE has a strong activity to inhibit inflammatory responses induced by LPS through the down-regulation of signal pathways related to TLR4-mediated inflammation in macrophages, and it is a promising natural resource for the development of functional health foods or medicines available for the prevention and treatment of inflammation-related diseases.

## Figures and Tables

**Table 1:** RNA preparation and quantitative real-time reverse transcription-polymerase chain reaction (real-time RT-PCR) analysis.

Gene	Forward	Reverse
TNF-α	AGC-CCC-CAG-TCT-GTA-TCC-TT	CTC-CCT-TTG-CAG-AAC-TCA-GG
IL-6	CCA-CGG-CCT-TCC-CTA-CTT-C	TTG-GGA-GTG-GTA-TCC-TCT-GTG-A
COX-2	CCA-CTT-CAA-GGG-AGT-CTG-GA	AGT-CAT-CTG-CTA-CGG-GAG-GA
iNOS	GTA-GTG-ACA-AGC-ACA-TTT-GG	GGC-TCC-ACT-TTT-CAC-TCT-GC

## Data Availability

Data are contained within the article.
